# A convolutional neural network to characterize mouse hindlimb foot strikes during voluntary wheel running

**DOI:** 10.3389/fbioe.2023.1206008

**Published:** 2023-06-13

**Authors:** Phillipe Huber, Brandon J. Ausk, K. Lionel Tukei, Steven D. Bain, Ted S. Gross, Sundar Srinivasan

**Affiliations:** Orthopaedic Science Laboratories, Department of Orthopaedics and Sports Medicine, Institute for Stem Cell and Regenerative Medicine, University of Washington, Seattle, WA, United States

**Keywords:** exercise, ground truth, machine learning, mice, voluntary wheel running

## Abstract

Voluntary wheel running (VWR) is widely used to study how exercise impacts a variety of physiologies and pathologies in rodents. The primary activity readout of VWR is aggregated wheel turns over a given time interval (most often, days). Given the typical running frequency of mice (∼4 Hz) and the intermittency of voluntary running, aggregate wheel turn counts, therefore, provide minimal insight into the heterogeneity of voluntary activity. To overcome this limitation, we developed a six-layer convolutional neural network (CNN) to determine the hindlimb foot strike frequency of mice exposed to VWR. Aged female C57BL/6 mice (22 months, n = 6) were first exposed to wireless angled running wheels for 2 h/d, 5 days/wk for 3 weeks with all VWR activities recorded at 30 frames/s. To validate the CNN, we manually classified foot strikes within 4800 1-s videos (800 randomly chosen for each mouse) and converted those values to frequency. Upon iterative optimization of model architecture and training on a subset of classified videos (4400), the CNN model achieved an overall training set accuracy of 94%. Once trained, the CNN was validated on the remaining 400 videos (accuracy: 81%). We then applied transfer learning to the CNN to predict the foot strike frequency of young adult female C57BL6 mice (4 months, n = 6) whose activity and gait differed from old mice during VWR (accuracy: 68%). In summary, we have developed a novel quantitative tool that non-invasively characterizes VWR activity at a much greater resolution than was previously accessible. This enhanced resolution holds potential to overcome a primary barrier to relating intermittent and heterogeneous VWR activity to induced physiological responses.

## Introduction

Voluntary wheel running (VWR) provides a low stress means of exploring how activity influences rodent physiological systems (Manzanares et al., 2018; Guo et al., 2020). Although VWR avoids the distress associated with enforced activity such as treadmill running, the intermittency and heterogeneity of uncontrolled voluntary activity hold potential to confound interpretation of data. For example, young mice exposed to 24 h VWR demonstrate weekly mean running distances that vary profoundly across time, age, and sex (Bartling et al., 2017). Not surprisingly, efforts to correlate activity levels (e.g., wheel turns) with exercise-induced adaptation (particularly where the observed response is modest, such as bone morphology) have not been successful (Schlecht et al., 2018).

Our interest in the VWR model arose from a desire to more accurately model the modest ability of exercise to increase bone mass in humans as compared to the robust bone adaptation observed in direct external bone loading models (Sun et al., 2017; Sato et al., 2020). In controlled skeletal loading models, high-resolution information describing the applied stimulus can be directly related to the adaptive response. From these studies, parameters such as peak strain and rest intervals have been associated with enhanced bone formation (Rubin and Lanyon, 1985; Gross et al., 1997). This approach is not viable for VWR, given the limited resolution of activity-related outcome measures.

VWR activity is typically quantified as wheel turns or distance run (derived from the wheel radius) in discrete time interval bins (usually 1 min or more) subsequently averaged across days or weeks (De Bono et al., 2006). Custom instrumented wheels have enabled high-resolution characterization of wheel running-induced gait kinematics and mechanical stimuli (Kitsukawa et al., 2011; Alvarez et al., 2012). However, these solutions require specialized equipment and instrumentation that are not commercially available and are only able to quantify a subset of activity within a study. We, therefore, drew inspiration from recent applications of machine learning and neural networks to biological problems and, specifically, efforts to quantify open-field behavior in mice (Sturman et al., 2020; Greener et al., 2022; Sheppard et al., 2022). Here, we describe a novel convolutional neural network (CNN) that enables classification of hindlimb foot strikes occurring within each 1-s interval throughout a VWR exposure.

## Methods

### *In vivo* voluntary wheel running

Aged female C57BL/6 mice (20 Mo, n = 6) were housed as a group per standard of care at the University of Washington. To familiarize mice with the wheel running apparatus (Goh and Ladiges, 2015), individual mice were placed in single cages each with one manually locked low-profile wireless running wheel contained within a light, sound-attenuated cabinet (Med Associates, Inc.) for 2 h/d beginning at 8 AM for three consecutive days (W–F). On the subsequent Monday, each mouse was exposed to its own unlocked running wheel for 2 h/d, 5 days/wk, for 3 weeks. All animals were euthanized without running on day 19 (i.e., Friday of wk 3; after 14 VWR exposures for each mouse). Upon completion of each wheel exposure, mice were returned to their group cages (at either 10 AM or 12 PM). Wheel turn counts (1 min bins) were recorded via wireless wheel software, and activity was recorded throughout each mouse’s 14 running wheel exposures using a near-IR video camera mounted on the cabinet’s interior roof (30 frames/s; 216,000 frames/mouse/VWR exposure to running wheels; Med Associates, Inc.).

The VWR experiment was then replicated in young adult female C57BL/6 mice (4 months, n = 6), with one exception (due to a calendar oversight, the mice received a total of 13, 2-h running wheel exposures).

### Human labeling of mouse hindlimb foot strikes

To manually classify foot strikes during VWR, we developed semi-automated software (MATLAB) to count the number of hindlimb foot strikes that occur every 1 s during mouse wheel running activity. For each of the six aged C57BL/6 mice, we randomly sampled 800, 1-s videos from within 2 h x 14 bouts of total VWR exposure (generating 4,800 1 s videos). Using slow-motion playback, we counted the number of hindlimb foot strikes within each 1-s video and converted that value to frequency. As there was no requirement for the mouse to be on a wheel, portions of the sampled videos also included periods where the mouse was not physically on the wheel or was on the wheel but not moving. The foot strike frequency for these conditions was classified as 0 Hz. Foot strikes were identically quantified for the young adult C57BL/6 mice (except that only 1000 1-s videos were sampled and classified). To establish a standard measure to contrast against the CNN model’s performance, we estimated human labeling accuracy. As no ground-truth exists for this dataset (used to assess machine learning accuracy vs. the real world (Shpilman et al., 2017)), we assessed how trained experienced and untrained users classified foot strike frequency. Experienced users were co-authors who contributed to developing foot strike count software development associated with the project. Untrained users were co-authors who had no prior experience estimating foot strikes from activity videos. Both experienced (n = 2; PH, SS) and untrained users (n = 2; EMG, BJA) viewed the same novel set of 100, 1-s videos randomly sampled from the overall aged female mouse video dataset (2 h × 14 days of activity x 6 mice) and used the semi-automated MATLAB program to classify foot strike during wheel running.

### A CNN to predict the foot strike frequency in aged (22 months) female C57BL/6 mice

**FIGURE 1:**
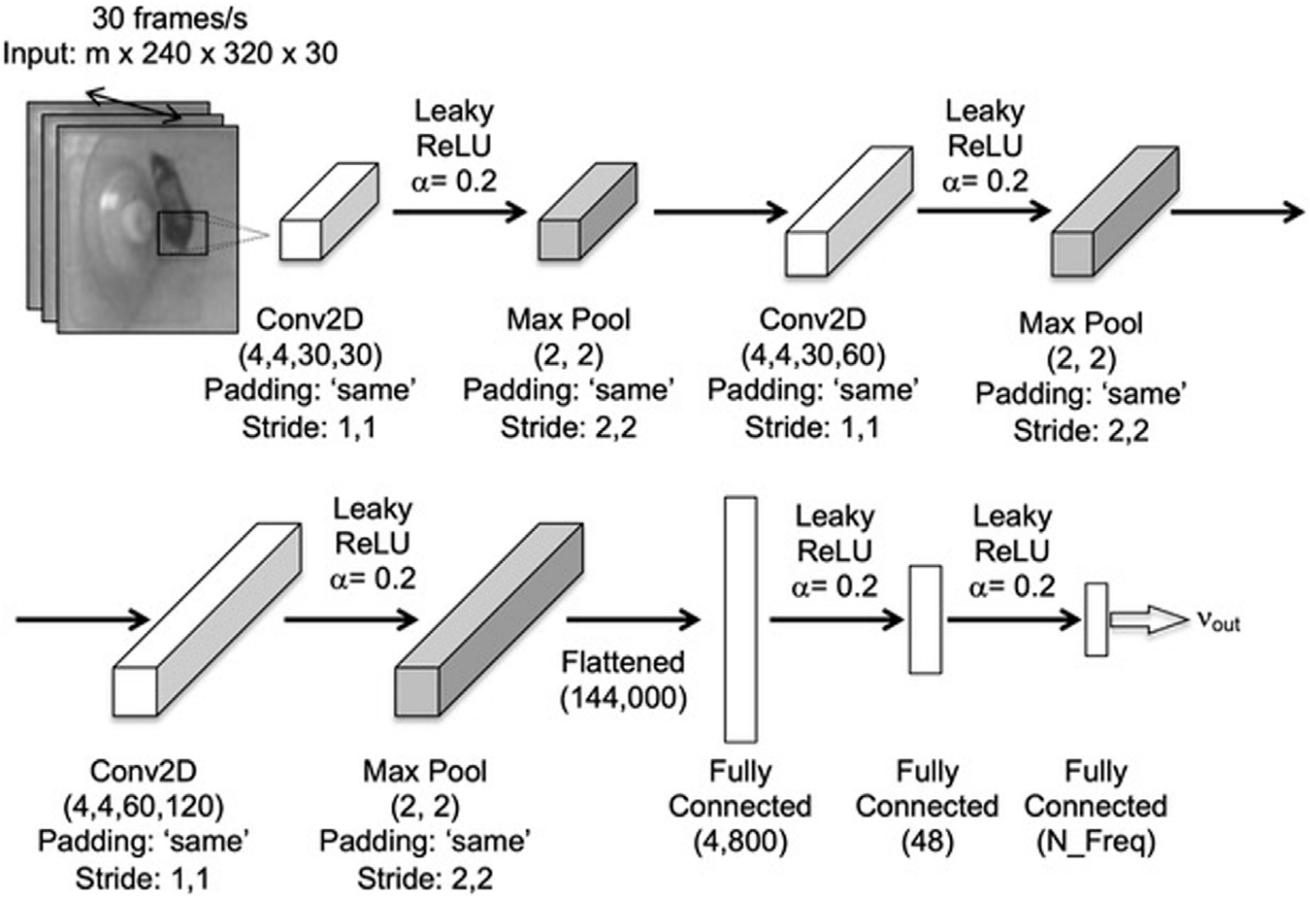
Schematic representation of the CNN model used to predict foot strike frequencies (v_out_), given a series of 30 image frames representing 1‐s of VWR. Model training was performed via a mini-batch (‘m’ videos) gradient descent. The CNN model implemented three Conv2D layers, each followed by max pooling where the same padding was used at each stage, with stride alternating between 1, 1 and 2, 2. Conv2D implemented a filter size of x, y, and z of n filters (beginning with 4, 4, 30, and 30). The final max pooling layer was flattened and fed a series of fully connected layers (number of neurons indicated) with the number of neurons in the final output layer (N_freq) specified as 8 for old mice and 11 for young adult mice (due to faster gait).

We framed the determination of foot strike frequencies via CNN as a multi-class (>three possible foot strike frequencies) supervised learning problem (human-labeled training data). To classify foot strike frequencies from mouse activity videos, we developed a CNN (Figure 1) using Python (v 3.6) and TensorFlow (v 1.14; CNN software is shared in a GitHub repository: https://github.com/osl-uw/cnn-foot-strike-frequency). The model incorporates three, 2-D convolutional layers (with leaky ReLU activations and max pooling layers), followed by three fully connected layers (with leaky ReLU activations (Maas et al., 2013)). The activations of the last of the fully connected layers are used to predict the foot strike frequency (in Hz), given a sequence of 30 image frames (240 × 320 pixels/frame) representing 1-s of VWR activity. For context, mouse foot strikes occur between 2.5 and 10 Hz during normal locomotion (Bellardita and Kiehn, 2015).

As the human classified data were not uniformly distributed across the foot strike frequency (Table 1, *p* < 0.001), stratified random sampling was implemented to split the data into training (90%) and test sets (10%). To train the model (and its parameters), the cross-entropy multi-class loss was minimized via a mini-batch gradient descent (Kingma and Ba, 2014). For this, the activations of the final layer were transformed via a softmax function and subsequently used to compute the multi-class cross-entropy loss. A stair-step learning rate decay (updated at the end of each training epoch) was also implemented for the training task. Initially, we augmented the training dataset (with 90% of 4800 resulting in 4320 1-s videos) via flipping (i.e., original, horizontal, vertical, and horizontal–vertical) without/with the addition of Gaussian noise (i.e., 4320 × 4 × 2) prior to testing against the remaining 10% of raw labeled data (480 1-s videos (Krizhevsky et al., 2012; Xu et al., 2017)). To assess whether augmentation was necessary, given that mice are primarily fixed in space during wheel running, an alternate model was trained on a separate non-augmented dataset (n = 4320, 1-s videos) and tested against separate raw labeled data (n = 480, 1-s videos).

**TABLE 1:** Foot strike frequency fractions in the human-labeled dataset for aged (n = 4,800 videos) and young (n = 1000 videos) female C57BL/6 mice. The data were not uniformly distributed across foot strike frequency (*p* < 0.001). Neural network hyperparameter settings: learning rate = 0.0001, decay rate = 0.95, and batch size = 36.

	0 Hz	1 Hz	2 Hz	3 Hz	4 Hz	5 Hz	6 Hz	7 Hz	8 Hz
**Aged**	68.6	1.7	3.7	5.6	8.3	8.6	3.4	0.1	0.0
**Young**	49.2	2.0	3.1	2.2	3.8	5.7	15.4	16.8	1.8

CNN models were separately trained on augmented and non-augmented datasets over 50 epochs (where each epoch involves traversing once over the entire training data set). Post-training, model accuracy was assessed via evaluation of the test dataset (i.e., naïve data not involved in training). As test accuracy was similar, regardless of augmentation, we used the less computationally intensive non-augmented trained model to predict the foot strike frequency within the 2 h/d x 14 days of activity videos for each aged mouse.

### Transfer learning to predict the foot strike frequency in young adult female C57BL/6 mice

Consistent with the literature, young adult mice qualitatively displayed differing gaits and speeds while running vs. the old mice (Tarantini et al., 2019). Preliminary analyses, not surprisingly, indicated that the CNN model trained upon old C57BL/6 mice activity data did not accurately predict the foot strike frequencies in young adult C57BL/6 mice (53% accuracy). To overcome this limitation, we used transfer learning to ‘transform’ the CNN model previously trained to the data distribution, representative of aged mice to that observed for young mice (Chan et al., 2020). As transfer learning re-used the pretrained CNN model, we were able to implement a minimal dataset compared to initial CNN model development (i.e., 1000 vs. 4800 videos). The labeled 1000 1-s videos obtained from young mice were split into train (n = 900) and test sets (n = 100) via stratified sampling. Using the same architecture (i.e., Figure 1), the CNN model’s parameters for all layers were first initialized to those of the model successfully trained against data from aged mice. Additional parameter training (100 epochs) was then initiated using data from young mice. Post-training, model accuracy was determined via prediction of the labeled raw test data.

### Statistical analysis

Contrasts of the labeled foot strike frequencies quantified for aged vs. young adult mice were performed via the Pearson’s chi-squared test of independence. The accuracy of the CNN model was evaluated via the assessment of predictions against labeled test data sets. Linear regression was implemented to examine the relationship between aggregate foot strikes predicted by the CNN model *versus* wheel turn counts aggregated over each 2-h running wheel exposure.

## Results

We first explored the reproducibility of the hindlimb foot strike frequency, as labeled by both experienced and untrained human users. Manual classification was performed at an average of ∼3 videos/min. For the classification dataset, the accuracy of classification between experienced users was 82.5% and the accuracy between untrained users was 75.1%.

**FIGURE 2:**
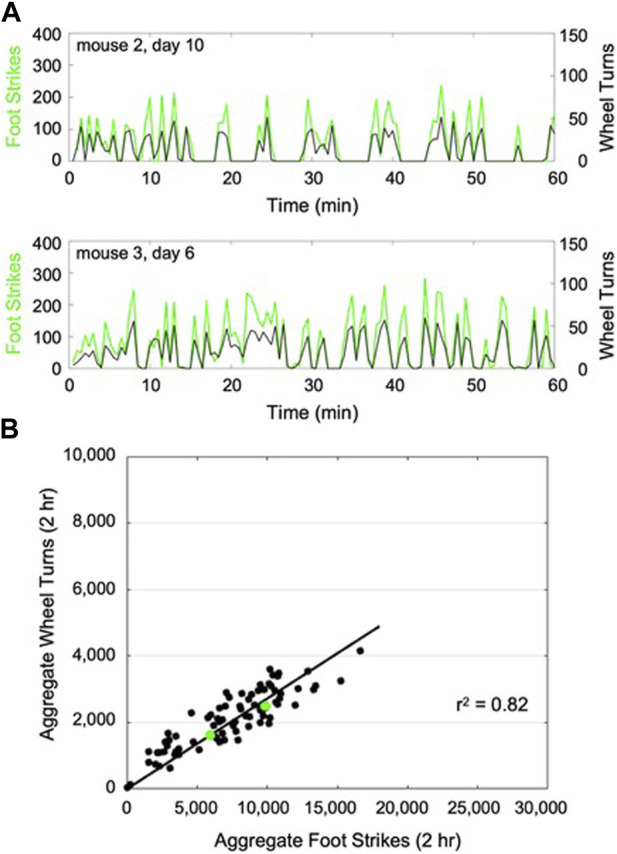
Representative plots of CNN predicted foot strikes and wheel turn counts for two representative aged mouse wheel exposures **(A)**, (discretized in 1 min bins). Across all aged mouse wheel exposures (14 bouts x 6 mice), cumulative predicted foot strikes per bout were highly correlated with cumulative wheel turn counts for that bout **(B)**, (*r* ^2^ = 0.82). The 2-h wheel exposures illustrated in **(A)** are highlighted in **(B)** (green).

When the CNN model was trained against data from aged C57BL/6 mice, model accuracy in classifying the training dataset was 100% for the augmented dataset and 94% for the non-augmented dataset. When evaluated against the test set, the model accuracy for the augmented dataset was 79% vs. 81% for the non-augmented data set. The non-augmented model was then used to predict foot strikes throughout each 2-h wheel running exposure. For a given wheel exposure, predicted foot strikes had a high level of temporal correspondence with wheel turn counts (Figure 2A). Across all mice and all wheel exposures, we found that predicted foot strikes were significantly correlated with aggregated wheel turn counts (*r* ^2^ = 0.82, *p* < 0.001; Figure 2B).

**FIGURE 3:**
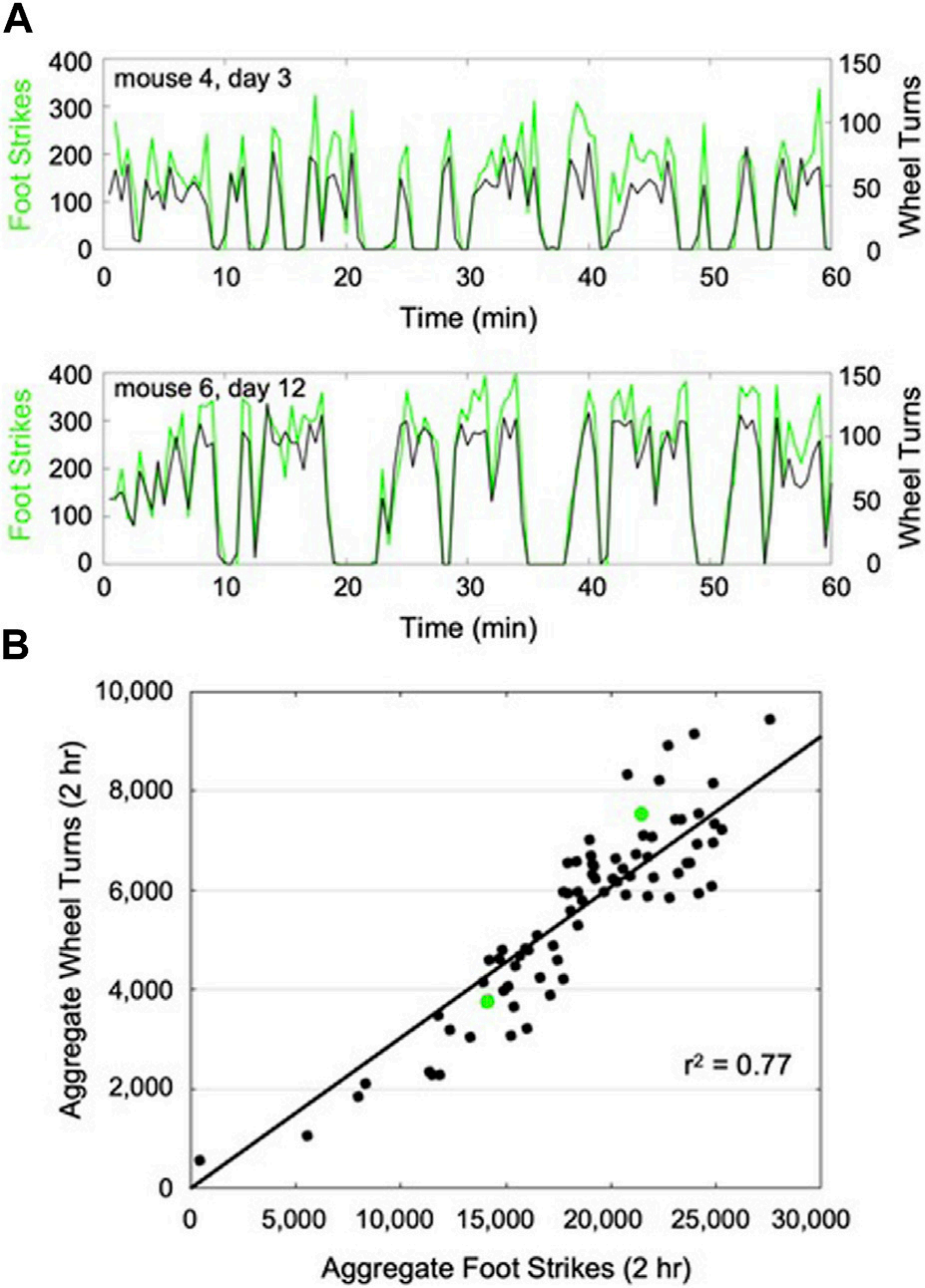
Representative plots of CNN predicted foot strikes and wheel turn counts for two representative young mouse wheel exposures **(A)**, (discretized in 1 min bins). Across all young adult mouse wheel exposures (13 bouts x 6 mice), cumulative predicted foot strikes per bout were highly correlated with cumulative wheel turn counts for that bout **(B)**, (*r* ^2^ = 0.77). The 2-h wheel exposures described in **(A)** are highlighted in **(B)** (green).

Following the implementation of transfer learning, the enhanced CNN was able to classify the young adult mouse train dataset at 98% accuracy. The enhanced CNN demonstrated 68% accuracy when applied to the young mouse test dataset. As with aged mice, CNN-derived foot strike counts temporally mirrored wheel turn counts across mice that demonstrated varied activity intermittency during wheel exposure, while cumulative foot strikes were significantly correlated with cumulative wheel turn counts (*r* ^2^ = 0.77, *p* < 0.001; Figure 3).

**FIGURE 4:**
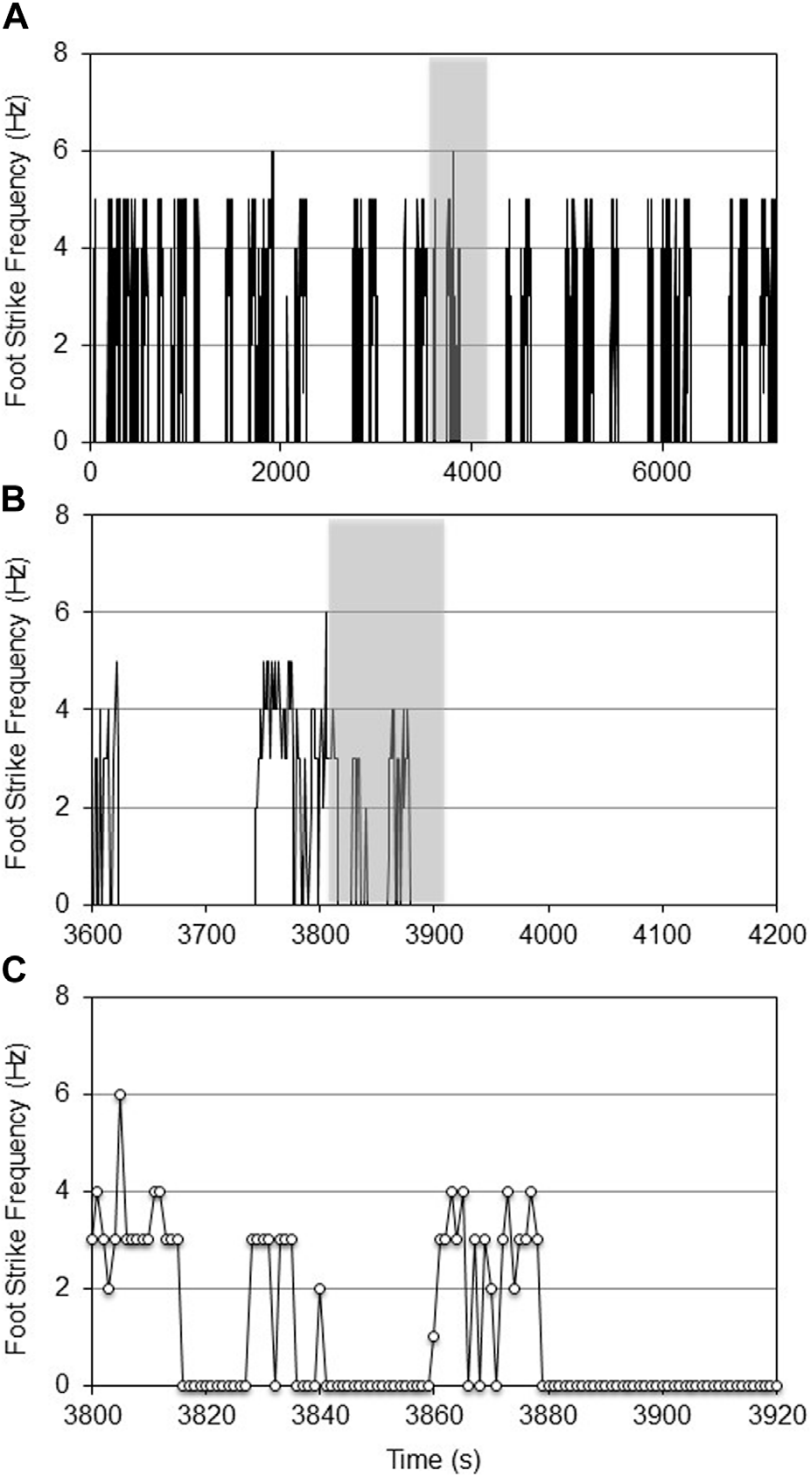
CNN predicted foot strike frequencies for a representative aged female mouse during a 2-h VWR exposure (d7 of 14, **(A)**). The 10-min shaded portion of Figure 4A is expanded in **(B)**, while the shaded 120-s portion of Figure 4B is expanded in **(C)**. At this temporal resolution, the intermittent heterogeneity of VWR was evident.

**FIGURE 5:**
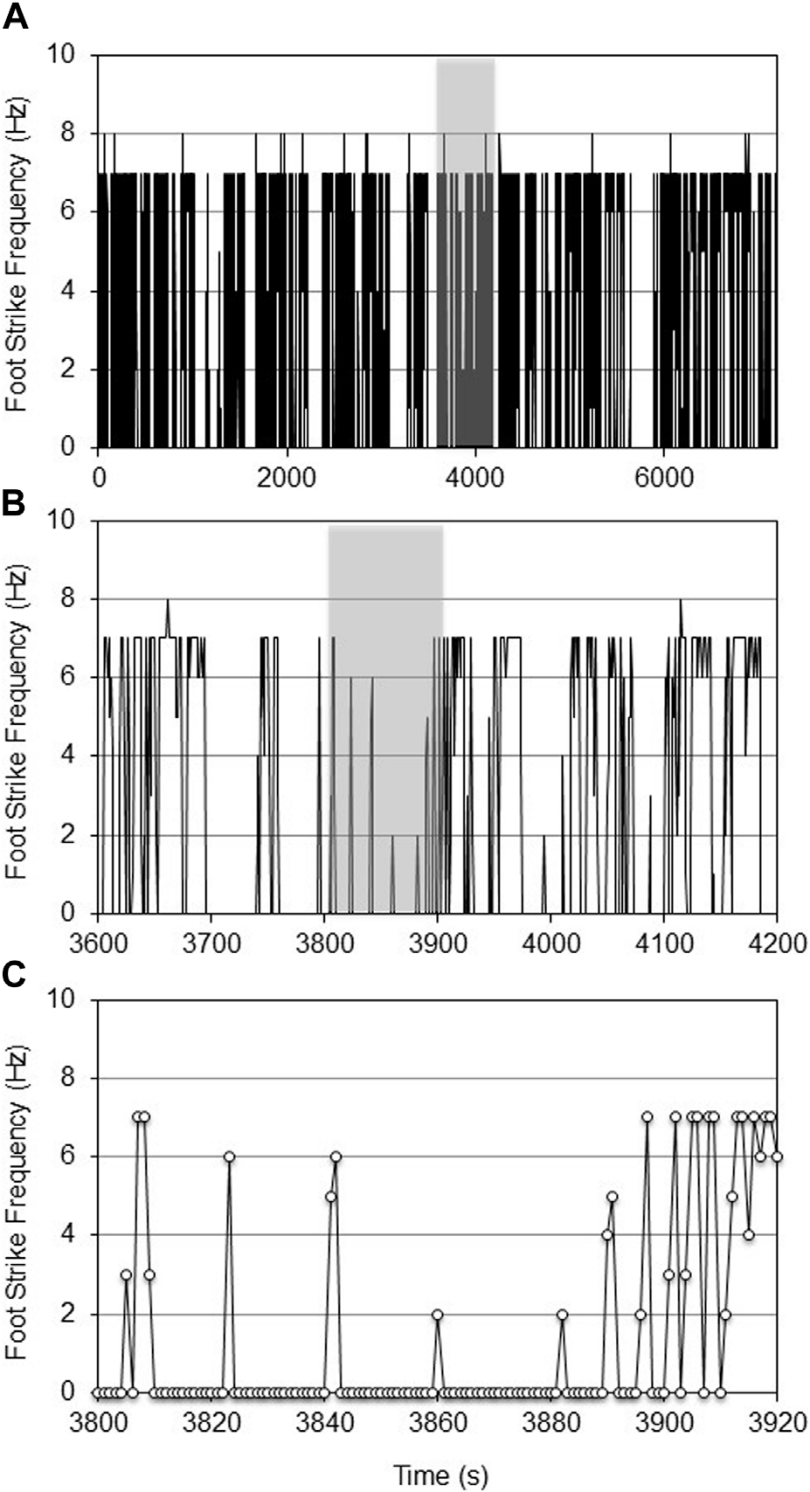
CNN predicted foot strike frequencies for a single young adult female mouse during 2-h VWR exposure (d7 of 13, **(A)**). The 10-min shaded portion of Figure 5A is expanded in **(B)**, while the shaded 120-s portion of Figure 5B is expanded in **(C)**. Increased activity was observed in younger mice vs. older mice (5A vs. 4A), as was expected from the literature, yet at 1 s resolution, activity was also clearly intermittent.

The age-specific, non-augmented CNN models were then used to quantify the foot strike frequency across the respective experimental datasets. Qualitatively, there were no repeatable patterns of wheel running activity. Wheel running was highly episodic with periods of variable length ‘rest’ (where the mouse was either resting, eating, or moving in the cage but not on the wheel). Foot strike frequency was also profoundly heterogeneous within and between episodes of wheel running (Figures 4, 5).

**FIGURE 6:**
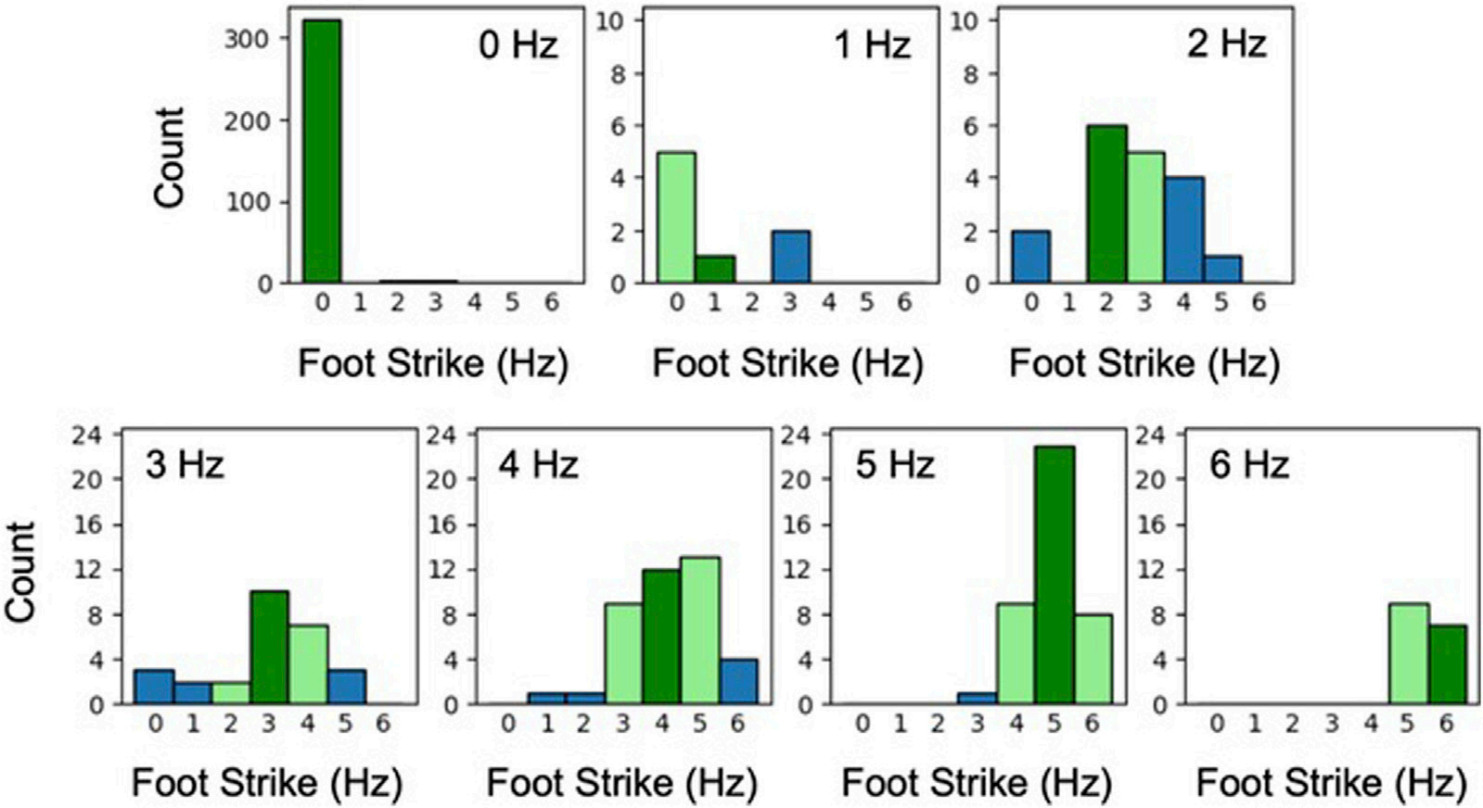
Histograms of CNN predicted foot strike frequency plotted separately for each user-labeled foot strike frequency (aged mouse dataset; 0–6 Hz). Dark green indicates when the CNN prediction matched labeled frequency, light green indicates a predicted frequency within 1 Hz of labeled frequency, and blue indicates >1 Hz difference between the predicted and labeled frequency. For example, the experienced user identified 40 videos as demonstrating 4-Hz foot strikes. For these same 40 videos, the CNN identified 12 as 4-Hz foot strikes and 34 of 40 (85%) as 3, 4, or 5 Hz.

Last, we explored CNN predictions of foot strikes within the aged mice test dataset as a function of user-identified foot strike frequency. The data predominantly contained 0-Hz wheel activity (69% of the total dataset), which was accurately identified by the CNN (99%, Figure 6). The CNN had difficulty precisely predicting both slower (1 and 2 Hz combined: 42%) and faster gait (3–6 Hz combined: 41%). However, when success criteria were loosened to accept CNN predictions within 1 Hz of labeled data, accuracy was elevated (1–2 Hz: 65%; 3–6 Hz: 88%).

## Discussion

We developed a six-layer CNN to non-invasively quantify mouse hindlimb foot strikes that occur within each 1-s interval throughout a 2-h VWR exposure. Our approach required two relatively inexpensive, commercially available pieces of equipment: an angled running wheel that fits within a standard mouse cage and a video activity-monitoring cabinet. We found that the CNN predicted foot strike frequency for old female C57BL/6 mice at accuracies approaching that of experienced humans. Transfer learning implemented via an additional smaller labeled dataset modestly enhanced the original model’s accuracy to predict the foot strike frequency in young adult female C57BL/6 mice.

The CNN model did not incorporate explicit mechanisms associated with classifying hindlimb foot strikes. For example, there was no assumption that there was a wheel or even a mouse visible within the video frame. Instead, input comprised a set of randomly selected 1-s videos that included mouse activity (or inactivity) on and off the wheel (Supplementary Figure S1 is a representative 1-s video containing five heel strikes). The CNN model foot strike frequency output was trained upon human-labeled foot strike frequency data. This strategy was consistent with typical development of CNNs, which are a class of deep neural networks commonly used for analyzing visual imagery (Anwar et al., 2018). Once trained, CNNs essentially function as black boxes (Bilbrey et al., 2020). To gain an initial understanding of how our CNN successfully performed the foot strike classification, we visualized activation at the end of each layer in the CNN (Supplementary Figures S2 and S3). Qualitatively, it appeared that the training dataset enabled the CNN model to distinguish outlines of the mouse, and, in particular, alterations in mouse hindquarter shape during VWR, which was previously observed in a recent machine learning assessment of mouse activity (Sheppard et al., 2022).

The old mouse training dataset enabled the CNN model to predict the foot strike frequency as accurately as the correspondence between two experienced humans (81% vs. 82.5%). However, given the literature demonstrating that young mice display different VWR behaviors than old mice (Manzanares et al., 2018; Bruns et al., 2020), a diminished accuracy when the CNN was used to classify the foot strike frequency in young mice was not surprising. We found that transfer learning enhanced the ability of the CNN initially trained on old mice to predict young mouse foot strike (+28% vs. original CNN). This improvement was achieved despite a secondary human-labeled data-set that was nearly 80% smaller than the initial training dataset. Although developing a *de novo* CNN, for every potential experimental condition (e.g., mouse weight, shape, coat color, and gait characteristics) would be an impediment to adoption of our approach, these data suggest that transfer learning on smaller datasets can overcome this limitation.

Recent studies have used CNN/machine learning in combination with commercial algorithms to characterize mouse gait and posture during open-field activity (Sturman et al., 2020; Sheppard et al., 2022). Closer to the challenge we addressed, machine learning has recently been used to quantify sleep stages in mice (Geuther et al., 2022). High-resolution quantification of mouse gait during VWR poses an additional barrier as the activity is spatially confined when gait is most rapid (e.g., when beginning to ambulate, mice move forward on the wheel, but once running ensues, they are spatially static until they decrease gait speed). Our approach is flexible as it provides a framework to use CNNs to quantify any gait-related behavior. The primarily limitation to this goal (which could include future interfacing with commercial software) is the ability to visualize and reproducibly label ‘gold-standard/ground-truths.’

There are additional limitations to our study. As often occurs with CNNs, ground-truth labels did not exist for the challenge we explored. We, therefore, developed our own dataset to train the CNN using foot strikes classified by one experienced user. This simplified approach was chosen as one of our goals was to explore the minimum necessary data labeling to enable an accurate CNN. We did explore whether the trained CNN would successfully predict foot strike frequency data labeled by an independent viewer (the second experienced user). For this preliminary test, the CNN did not require additional transfer learning (i.e., the CNN was similarly accurate in predicting data labeled by both experienced observers; data not shown).

Second, there are a high number of variables underlying the mouse behavior during VWR that hold potential to undermine the relation between foot strike quantification and wheel turns (or distance traveled). For example, mice frequently exhibit a burst of running, and then jump off the wheel, which continues to rotate due to angular momentum. Additionally, the speed, direction, and duration of each activity burst are highly heterogeneous, even within a few minutes. Despite these limitations, cumulative CNN-quantified foot strikes predicted approximately 80% of the variability of wheel turn counts within each 2-h activity bout.

Finally, we did not focus on optimizing CNN accuracy or identifying trends within the foot strike datasets as our granular analysis (Figure 6) suggested there is substantial room for improvement of CNN accuracy. For example, we believe the inaccuracy at 1 and 2 Hz arose from both physiologic (mouse walking gait is generally classified to begin at 2.5 Hz (Bellardita and Kiehn, 2015)) and labeling limitations (a single foot strike in a 1-s video was observed at the end or beginning of the frame grab, representing the initiation or cessation of activity, respectively). This might be addressed by altering the framework we used to label test data from discrete videos to enable continuous sampling. The temporal signature of foot strikes within a given wheel exposure emphasizes the benefit that might be achieved with this enhancement (only limited by imaging resolution). Ultimately, we believe that CNN accuracy would be enhanced by a combination of increased training and testing dataset size (as augmented datasets appeared to have minimal benefit), enriching the ratio of activity vs. non-activity occurrences within labeled videos, architectural refinements (e.g., reframing as an ordinal regression problem, hyper parameter tuning), and more efficient image pre-processing (to enhance computation speed).

Despite these limitations, we were able to non-invasively resolve hindlimb foot strikes during VWR at 1-s resolution for the first time. Even cursory observation of these gait ‘signatures’ revealed an extremely rich dataset characterized by heterogeneity between mice and across wheel exposures for the same mouse. Like humans, some mice demonstrated consistently greater daily foot strike counts than other mice, while some mice were characterized by variability within and across days. Unlike low-resolution measures such as wheel turn counts, which have been shown to be minimally related to induced tissue adaptation (Schlecht et al., 2018), we believe the complex heterogeneity of foot strikes holds substantial potential to identify those aspects of locomotory behavior that underlie adaptation induced by VWR. For example, in the context of bone mechanotransduction, the foot strike dataset could be explored to generate hypotheses regarding what aspect of behavior on the wheel (e.g., minimum foot strikes within a given time period, followed by sufficient rest intervals (Srinivasan et al., 2002; Srinivasan et al., 2015)) is correlated with skeletal adaptation. Given the variability and the size of the resulting datasets, development of unbiased data mining strategies will likely be required to relate VWR gait signatures to adaptation of the cardiovascular, musculoskeletal, or central nervous systems.

## Conclusion

We developed a CNN that accurately predicted hindlimb foot strikes at 1-s resolution throughout a voluntary wheel running exposure. This foot strike resolution holds potential to overcome a primary barrier that impairs the ability to relate intermittent heterogeneous wheel running activity to induced physiological responses. More broadly, we believe that this platform could be readily extended to quantify any gait-related behavior, for which ground-truth labeling can be estimated.

## Data Availability

The raw data supporting the conclusion of this article will be made available by the authors, without undue reservation.

## References

[B1] Alvarez M. L. Khosroheidari M. Kanchi Ravi R. Distefano J. K. (2012). Comparison of protein, microRNA, and mRNA yields using different methods of urinary exosome isolation for the discovery of kidney disease biomarkers. Kidney Int. 82, 1024–1032. 10.1038/ki.2012.256 22785172

[B2] Anwar S. M. Majid M. Qayyum A. Awais M. Alnowami M. Khan M. K. (2018). Medical image analysis using convolutional neural networks: A review. J. Med. Syst. 42 (11), 226. 10.1007/s10916-018-1088-1 30298337

[B3] Bartling B. Al-Robaiy S. Lehnich H. Binder L. Hiebl B. Simm A. (2017). Sex-related differences in the wheel-running activity of mice decline with increasing age. Exp. Gerontol. 87, 139–147. 10.1016/j.exger.2016.04.011 27108181

[B4] Bellardita C. Kiehn O. (2015). Phenotypic characterization of speed-associated gait changes in mice reveals modular organization of locomotor networks. Curr. Biol. 25 (11), 1426–1436. 10.1016/j.cub.2015.04.005 25959968 PMC4469368

[B5] Bilbrey J. A. Heindel J. P. Schram M. Bandyopadhyay P. Xantheas S. S. Choudhury S. (2020). A look inside the black box: Using graph-theoretical descriptors to interpret a Continuous-Filter Convolutional Neural Network (CF-CNN) trained on the global and local minimum energy structures of neutral water clusters. J. Chem. Phys. 153 (2), 024302. 10.1063/5.0009933 32668919

[B6] Bruns D. R. Yusifova M. Marcello N. A. Green C. J. Walker W. J. Schmitt E. E. (2020). The peripheral circadian clock and exercise: Lessons from young and old mice. J. Circadian Rhythms 18, 7. 10.5334/jcr.201 33384723 PMC7757608

[B7] Chan H. P. Samala R. K. Hadjiiski L. M. Zhou C. (2020). Deep learning in medical image analysis. Adv. Exp. Med. Biol. 1213, 3–21. 10.1007/978-3-030-33128-3_1 32030660 PMC7442218

[B8] De Bono J. P. Adlam D. Paterson D. J. Channon K. M. (2006). Novel quantitative phenotypes of exercise training in mouse models. Am. J. Physiol. Regul. Integr. Comp. Physiol. 290 (4), R926–R934. 10.1152/ajpregu.00694.2005 16339385

[B9] Geuther B. Chen M. Galante R. J. Han O. Lian J. George J. (2022). High-throughput visual assessment of sleep stages in mice using machine learning. Sleep 45 (2), zsab260. 10.1093/sleep/zsab260 34718812 PMC8842275

[B10] Goh J. Ladiges W. (2015). Voluntary wheel running in mice. Curr. Protoc. Mouse Biol. 5 (4), 283–290. 10.1002/9780470942390.mo140295 26629772 PMC4686373

[B11] Greener J. G. Kandathil S. M. Moffat L. Jones D. T. (2022). A guide to machine learning for biologists. Nat. Rev. Mol. Cell Biol. 23 (1), 40–55. 10.1038/s41580-021-00407-0 34518686

[B12] Gross T. S. Edwards J. L. McLeod K. J. Rubin C. T. (1997). Strain gradients correlate with sites of periosteal bone formation. J. Bone Min. Res. 12 (6), 982–988. 10.1359/jbmr.1997.12.6.982 9169359

[B13] Guo S. Huang Y. Zhang Y. Huang H. Hong S. Liu T. (2020). Impacts of exercise interventions on different diseases and organ functions in mice. J. Sport Health Sci. 9 (1), 53–73. 10.1016/j.jshs.2019.07.004 31921481 PMC6943779

[B14] Kingma D. P. Ba J. (2014). Adam: A method for stochastic optimization. arXiv preprint arXiv:14126980.

[B15] Kitsukawa T. Nagata M. Yanagihara D. Tomioka R. Utsumi H. Kubota Y. (2011). A novel instrumented multipeg running wheel system, Step-Wheel, for monitoring and controlling complex sequential stepping in mice. J. Neurophysiol. 106 (1), 479–487. 10.1152/jn.00139.2011 21525375 PMC3129737

[B16] Krizhevsky A. Sutskever I. Hinton G. (2012). ImageNet classification with deep convolutional neural networks. Neural Inf. Process. Syst. 25.

[B17] Maas A. L. Hannun A. Y. Ng A. Y. (2013). “Rectifier nonlinearities improve neural network acoustic models,” in ICML workshop on deep learning for audio, speech, and language processing.

[B18] Manzanares G. Brito-da-Silva G. Gandra P. G. (2018). Voluntary wheel running: Patterns and physiological effects in mice. Braz J. Med. Biol. Res. 52 (1), e7830. 10.1590/1414-431x20187830 30539969 PMC6301263

[B19] Rubin C. T. Lanyon L. E. (1985). Regulation of bone mass by mechanical strain magnitude. Calcif. Tissue Int. 37 (4), 411–417. 10.1007/bf02553711 3930039

[B20] Sato T. Verma S. Andrade C. D. C. Omeara M. Campbell N. Wang J. S. (2020). A FAK/HDAC5 signaling axis controls osteocyte mechanotransduction. Nat. Commun. 11 (1), 3282. 10.1038/s41467-020-17099-3 32612176 PMC7329900

[B21] Schlecht S. H. Ramcharan M. A. Yang Y. Smith L. M. Bigelow E. M. Nolan B. T. (2018). Differential adaptive response of growing bones from two female inbred mouse strains to voluntary cage-wheel running. JBMR Plus 2 (3), 143–153. 10.1002/jbm4.10032 30283899 PMC6124195

[B22] Sheppard K. Gardin J. Sabnis G. S. Peer A. Darrell M. Deats S. (2022). Stride-level analysis of mouse open field behavior using deep-learning-based pose estimation. Cell Rep. 38 (2), 110231. 10.1016/j.celrep.2021.110231 35021077 PMC8796662

[B23] Shpilman A. Boikiy D. Polyakova M. Kudenko D. Burakov A. Nadezhdina E. (2017). “Deep learning of cell classification using microscope images of intracellular microtubule networks,” in 16th IEEE international conference on machine learning and applications (Cancun), 1–6.

[B24] Srinivasan S. Ausk B. J. Bain S. D. Gardiner E. M. Kwon R. Y. Gross T. S. (2015). Rest intervals reduce the number of loading bouts required to enhance bone formation. Med. Sci. Sports Exerc 47 (5), 1095–1103. 10.1249/mss.0000000000000509 25207932 PMC4368504

[B25] Srinivasan S. Weimer D. A. Agans S. C. Bain S. D. Gross T. S. (2002). Low-magnitude mechanical loading becomes osteogenic when rest is inserted between each load cycle. J. Bone Min. Res. 17 (9), 1613–1620. 10.1359/jbmr.2002.17.9.1613 PMC1435731 12211431

[B26] Sturman O. von Ziegler L. Schlappi C. Akyol F. Privitera M. Slominski D. (2020). Deep learning-based behavioral analysis reaches human accuracy and is capable of outperforming commercial solutions. Neuropsychopharmacology 45 (11), 1942–1952. 10.1038/s41386-020-0776-y 32711402 PMC7608249

[B27] Sun D. Brodt M. D. Zannit H. M. Holguin N. Silva M. J. (2017). Evaluation of loading parameters for murine axial tibial loading: Stimulating cortical bone formation while reducing loading duration. J. Orthop. Res. 36, 682–691. 10.1002/jor.23727 28888055 PMC5839947

[B28] Tarantini S. Yabluchanskiy A. Fulop G. A. Kiss T. Perz A. O'Connor D. (2019). Age-related alterations in gait function in freely moving male C57bl/6 mice: Translational relevance of decreased cadence and increased gait variability. J. Gerontol. A Biol. Sci. Med. Sci. 74 (9), 1417–1421. 10.1093/gerona/gly242 30383221 PMC6696715

[B29] Xu K. Roussel P. Csapo T. G. Denby B. (2017). Convolutional neural network-based automatic classification of midsagittal tongue gestural targets using B-mode ultrasound images. J. Acoust. Soc. Am. 141 (6), EL531–EL537. 10.1121/1.4984122 28618815

