# Supplementary figures and images for: A convolutional neural network to characterize mouse hindlimb foot strikes during voluntary wheel running
Source: Front Bioeng Biotechnol. 2023 Jun 13;11:1206008. doi: 10.3389/fbioe.2023.1206008 (PMC10299834; doi:10.3389/fbioe.2023.1206008)

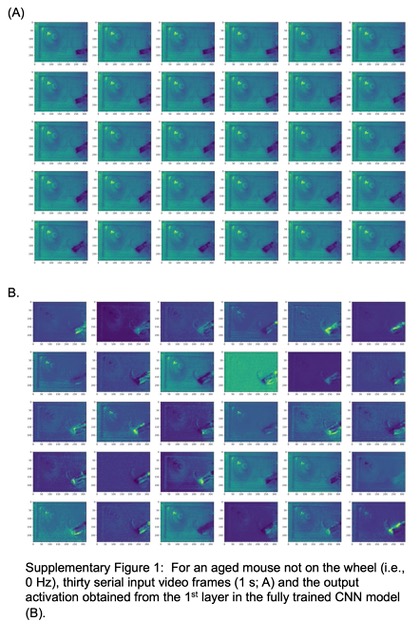

Supplement: Supplementary file 1 [file Image1.JPEG]

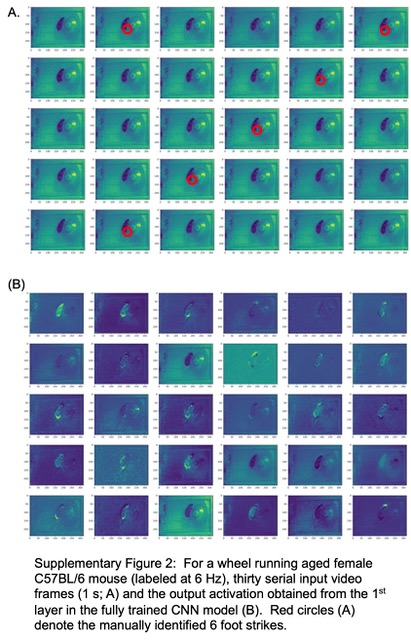

Supplement: Supplementary file 2 [file Image2.JPEG]
